# 6-Amino-9*H*-purine-1,7-diium bis­(4-methyl­benzene­sulfonate) monohydrate

**DOI:** 10.1107/S1600536810000413

**Published:** 2010-01-09

**Authors:** Zhi-Qiang Xiong, Yun-Long Ai, Hui-Liang Wen

**Affiliations:** a Center of Analysis and Testing, Nanchang Hangkong University, Nanchang 330063, People’s Republic of China; b State Key Laboratory of Food Science and Technology, Nanchang University, Nanchang 330047, People’s Republic of China

## Abstract

The asymmetric unit of the title compound, C_5_H_7_N_5_^2+^·2C_7_H_7_O_3_S^−^·H_2_O, consists of one diprotonated adeninium cation, two *p*-toluene­sulfonic acid anions and one water mol­ecule. In the crystal, the cations and anions are connected through N—H⋯O hydrogen bonds forming *R*_2_^2^(8) and *R*_2_^2^(9) graph-set motifs. The solvent water mol­ecule links cations and anions through O—H⋯O and N—H⋯O hydrogen bonds, generating a two-dimensional layer parallel to (10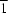).

## Related literature

For biological activity of purine and its derivatives, see: Barral *et al.* (2006 ▶); Sridhar & Ravikumar (2007 ▶); Sridhar *et al.* (2009 ▶); Xing *et al.* (2008 ▶). For hydrogen-bonding motifs, see: Etter (1990 ▶); Bernstein *et al.* (1995 ▶).
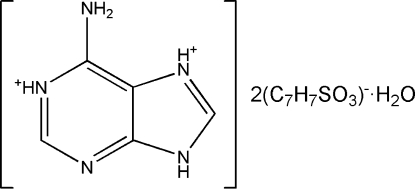


## Experimental

### 

#### Crystal data

C_5_H_7_N_5_^2+^·2C_7_H_7_O_3_S^−^·H_2_O
*M* *_r_* = 497.54
Monoclinic, 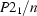

*a* = 16.2462 (11) Å
*b* = 6.0370 (4) Å
*c* = 22.7390 (15) Å
β = 90.625 (1)°
*V* = 2230.1 (3) Å^3^
*Z* = 4
Mo *K*α radiation
μ = 0.29 mm^−1^
*T* = 296 K
0.31 × 0.21 × 0.21 mm

#### Data collection

Bruker APEXII CCD diffractometer
Absorption correction: multi-scan (*SADABS*; Bruker, 2006 ▶) *T*_min_ = 0.915, *T*_max_ = 0.942
16652 measured reflections
4153 independent reflections
3452 reflections with *I* > 2σ(*I*)
*R*_int_ = 0.100

#### Refinement

*R*[*F* ^2^ > 2σ(*F* ^2^)] = 0.040
*wR*(*F* ^2^) = 0.120
*S* = 1.05
4153 reflections
300 parameters
H-atom parameters constrained
Δρ_max_ = 0.46 e Å^−3^
Δρ_min_ = −0.31 e Å^−3^

### 

Data collection: *APEX2* (Bruker, 2006 ▶); cell refinement: *SAINT* (Bruker, 2006 ▶); data reduction: *SAINT*; program(s) used to solve structure: *SHELXS97* (Sheldrick, 2008 ▶); program(s) used to refine structure: *SHELXL97* (Sheldrick, 2008 ▶); molecular graphics: *ORTEPIII* (Burnett & Johnson, 1996 ▶), *ORTEP-3 for Windows* (Farrugia, 1997 ▶) and *CAMERON* (Pearce *et al.*, 2000 ▶); software used to prepare material for publication: *SHELXTL* (Sheldrick, 2008 ▶).

## Supplementary Material

Crystal structure: contains datablocks global, I. DOI: 10.1107/S1600536810000413/dn2513sup1.cif

Structure factors: contains datablocks I. DOI: 10.1107/S1600536810000413/dn2513Isup2.hkl

Additional supplementary materials:  crystallographic information; 3D view; checkCIF report

## Figures and Tables

**Table 1:** Hydrogen-bond geometry (Å, °)

*D*—H⋯*A*	*D*—H	H⋯*A*	*D*⋯*A*	*D*—H⋯*A*
N1—H1*A*⋯O4	0.86	1.95	2.813 (2)	178
N1—H1*B*⋯O3	0.86	1.97	2.805 (2)	163
N2—H2*A*⋯O5	0.86	1.84	2.694 (2)	178
N4—H4⋯O7^i^	0.86	1.80	2.653 (2)	170
N5—H5*A*⋯O1	0.86	2.09	2.884 (3)	152
N5—H5*A*⋯O3	0.86	2.43	3.149 (3)	141
O7—H1*W*⋯O6^ii^	0.84	1.93	2.762 (2)	176
O7—H2*W*⋯O2	0.83	2.04	2.815 (2)	156

Symmetry codes: (i) 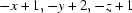; (ii) 
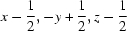
.

**Fig. 1.**
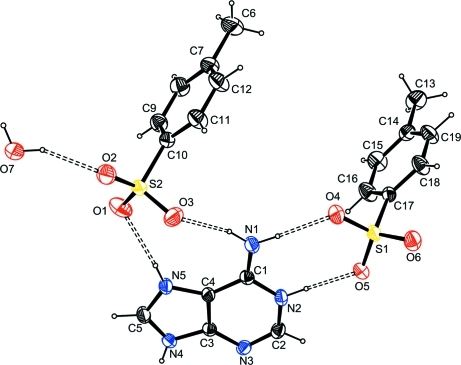
: A view of the structure of (I), showing the atom-numbering scheme. Displacement ellipsoids are drawn at the 30% probability level and H atoms are shown as small spheres of arbitrary radii. Hydrogen bonds are shown as dashed lines.

**Fig. 2.**
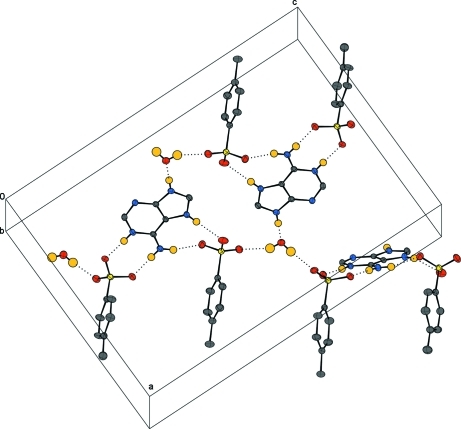
: Partial packing view of the title compound, viewed down the b axis. Hydrogen bonds are shown as dashed lines.

## supplementary crystallographic information

### Comment

Purin and its derivatives, as one kind of important nucleobase compounds, are essential for understanding many mechanisms of basic importance in the biological process (Xing *et al.*, 2008), and belongs to a group of cytokinin-derived compounds which are indispensable for plant growth. The concept of the acyclic nucleoside phosphonate (ANPs) has been used to design chain terminators for antiviral and therapy proved to be valid (Barral *et al.*, 2006). In C_5_H_6_N_5_^+^.C_8_H_7_O_2_^–^.C_8_H_8_O_2_^–^.H_2_O and C_5_H_6_N_5_^+^.C_4_H_3_O_4_^–^.H_2_O compounds, the adeninium cations form N-H···O hydrogen bonds with their anion counterparts and adeninium-adeninium self-association base pairs (Sridhar *et al.*, 2007). In C_5_H_7_N_5_^2+^.0.5C_2_O_4_^2–^.2Cl^– ^compound, adenine is doubly protonated, while in C_5_H_6_N_5_^+^.C_2_HO_4_^–^.0.5C_2_H_2_O_4_.H_2_O compound, adenine is monoprotonated (Sridhar *et al.*, 2009).

In the title compound, C_5_H_7_N_5_^2+^.2C_7_H_7_SO_3_^–^.H_2_O, the asymmetric unit contains one diprotonated adeninium cation, two *p*-toluenesulfonic acid anions and one water molecule. The two anions are connected to the purin molecule through N-H···O hydrogen bonds building R_2_^2^(8) and R_2_^2^(9) graph set motifs (Table 1, Fig.1) (Etter, 1990; Bernstein *et al.*, 1995) . In the same asymmetric unit, the water molecule is connected through O-H···O hydrogen bond to one of the *p*-toluenesulfonic acid (Table 1, Fig. 1).

Futhermore The water links cation and anion through O-H···O and N-H···O hydrogen bonds forming a R66(20) graph set motif and buiding a two dimensional layer parallel to the (1 0 -1) plane (Fig. 2, Table 1).

In the 9*H*-purin-6-amine molecule, all atoms are coplanar, the dihedral angles between the plane of the 9*H*-purin-6-amine and the benzene rings of the p-toluenesulfonate anions are 87.78 (5)° and 87.15 (5)°, respectively, indicating that the 9*H*-purin-6-amine is almost perpendicular to the two benzene rings.

### Experimental

A mixture of purin-6-amine (10.0 mmol) and toluene sulfonic acid (20.0 mmol) was dissolved in ethanol (40 ml) in batches over a period of 2 h under reflux, heating was continued for 1 h. The mixture was cooled to room temperature and separated, the solvent of the organic phase was removed and the residue recrystallized with ethyl acetate. Yellow crystals of the title compound suitable for X-ray diffraction analysis were obtained after several days. Yield 77.3%.

### Refinement

The water H atoms were located in a difference Fourier map but were included in fixed positions in riding-model approximation with the O—H distances in the range 0.8252–0.8381Å and *U*_iso_(H) = 1.5*U*_eq_(O); all other H atoms were placed in geometrically idealized positions with C<–H(methylene) = 0.96 Å, C–H(aromatic) = 0.93 Å, N—H = 0.86 Å, and *U*_iso_(H) = 1.2 *U*_eq_(C,N).

### Crystal data

**Table:**

C_5_H_7_N_5_^2+^·2C_7_H_7_O_3_S^−^·H_2_O	*F*(000) = 1040
*M**_r_* = 497.54	*D*_x_ = 1.482 Mg m^−^^3^
Monoclinic, *P*2_1_/*n*	Mo *K*α radiation, λ = 0.71073 Å
Hall symbol: -P 2yn	Cell parameters from 7679 reflections
*a* = 16.2462 (11) Å	θ = 2.5–28.1°
*b* = 6.0370 (4) Å	µ = 0.29 mm^−^^1^
*c* = 22.7390 (15) Å	*T* = 296 K
β = 90.625 (1)°	Block, yellow
*V* = 2230.1 (3) Å^3^	0.31 × 0.21 × 0.21 mm
*Z* = 4	

### Data collection

**Table:**

Bruker APEXII CCD diffractometer	4153 independent reflections
Radiation source: fine-focus sealed tube	3452 reflections with *I* > 2σ(*I*)
graphite	*R*_int_ = 0.100
φ and ω scans	θ_max_ = 25.5°, θ_min_ = 2.5°
Absorption correction: multi-scan (*SADABS*; Bruker, 2006)	*h* = −19→19
*T*_min_ = 0.915, *T*_max_ = 0.942	*k* = −7→7
16652 measured reflections	*l* = −27→26

### Refinement

**Table:**

Refinement on *F*^2^	Primary atom site location: structure-invariant direct methods
Least-squares matrix: full	Secondary atom site location: difference Fourier map
*R*[*F*^2^ > 2σ(*F*^2^)] = 0.040	Hydrogen site location: inferred from neighbouring sites
*wR*(*F*^2^) = 0.120	H-atom parameters constrained
*S* = 1.05	*w* = 1/[σ^2^(*F*_o_^2^) + (0.059*P*)^2^ + 0.6101*P*] where *P* = (*F*_o_^2^ + 2*F*_c_^2^)/3
4153 reflections	(Δ/σ)_max_ < 0.001
300 parameters	Δρ_max_ = 0.46 e Å^−^^3^
0 restraints	Δρ_min_ = −0.31 e Å^−^^3^

### Special details

**Table:**

Geometry. All esds (except the esd in the dihedral angle between two l.s. planes) are estimated using the full covariance matrix. The cell esds are taken into account individually in the estimation of esds in distances, angles and torsion angles; correlations between esds in cell parameters are only used when they are defined by crystal symmetry. An approximate (isotropic) treatment of cell esds is used for estimating esds involving l.s. planes.
Refinement. Refinement of *F*^2^ against ALL reflections. The weighted *R*-factor *wR* and goodness of fit *S* are based on *F*^2^, conventional *R*-factors *R* are based on *F*, with *F* set to zero for negative *F*^2^. The threshold expression of *F*^2^ > σ(*F*^2^) is used only for calculating *R*-factors(gt) *etc*. and is not relevant to the choice of reflections for refinement. *R*-factors based on *F*^2^ are statistically about twice as large as those based on *F*, and *R*- factors based on ALL data will be even larger.

### Fractional atomic coordinates and isotropic or equivalent isotropic displacement parameters (Å^2^)

**Table:**

	*x*	*y*	*z*	*U*_iso_*/*U*_eq_	
C1	0.58230 (11)	0.6806 (3)	0.70038 (8)	0.0345 (4)	
C2	0.68007 (12)	0.9106 (3)	0.75121 (9)	0.0424 (5)	
H2	0.7096	0.9311	0.7860	0.051*	
C3	0.64254 (11)	1.0129 (3)	0.66210 (8)	0.0342 (4)	
C4	0.58820 (11)	0.8401 (3)	0.65534 (8)	0.0342 (4)	
C5	0.58704 (13)	1.0362 (3)	0.57478 (9)	0.0439 (5)	
H5	0.5744	1.0860	0.5371	0.053*	
C6	0.12147 (18)	0.2967 (6)	0.73440 (13)	0.0827 (9)	
H6A	0.1439	0.2918	0.7736	0.124*	
H6B	0.0967	0.1566	0.7250	0.124*	
H6C	0.0806	0.4113	0.7319	0.124*	
C7	0.18958 (15)	0.3450 (4)	0.69138 (11)	0.0578 (6)	
C8	0.19569 (15)	0.5466 (4)	0.66313 (11)	0.0605 (6)	
H8	0.1573	0.6566	0.6709	0.073*	
C9	0.25782 (14)	0.5885 (4)	0.62340 (10)	0.0523 (5)	
H9	0.2609	0.7254	0.6048	0.063*	
C10	0.31520 (12)	0.4262 (3)	0.61154 (9)	0.0410 (5)	
C11	0.30975 (16)	0.2236 (4)	0.63958 (11)	0.0577 (6)	
H11	0.3479	0.1129	0.6318	0.069*	
C12	0.24765 (16)	0.1866 (4)	0.67901 (12)	0.0653 (7)	
H12	0.2449	0.0501	0.6979	0.078*	
C13	0.30034 (16)	0.4904 (5)	1.01182 (12)	0.0714 (8)	
H13A	0.2502	0.4442	0.9928	0.107*	
H13B	0.2984	0.6471	1.0189	0.107*	
H13C	0.3066	0.4135	1.0485	0.107*	
C14	0.37220 (13)	0.4378 (4)	0.97288 (9)	0.0470 (5)	
C15	0.40420 (16)	0.5906 (4)	0.93554 (12)	0.0622 (7)	
H15	0.3813	0.7317	0.9344	0.075*	
C16	0.46944 (16)	0.5434 (4)	0.89932 (11)	0.0595 (6)	
H16	0.4907	0.6524	0.8749	0.071*	
C17	0.50300 (12)	0.3336 (3)	0.89954 (8)	0.0374 (4)	
C18	0.47223 (15)	0.1783 (4)	0.93719 (10)	0.0558 (6)	
H18	0.4949	0.0369	0.9382	0.067*	
C19	0.40759 (15)	0.2304 (4)	0.97374 (11)	0.0599 (6)	
H19	0.3876	0.1236	0.9994	0.072*	
N1	0.53750 (11)	0.5001 (3)	0.69920 (7)	0.0431 (4)	
H1A	0.5384	0.4109	0.7287	0.052*	
H1B	0.5072	0.4708	0.6689	0.052*	
N2	0.63051 (10)	0.7299 (3)	0.74765 (7)	0.0397 (4)	
H2A	0.6296	0.6412	0.7772	0.048*	
N3	0.68966 (10)	1.0571 (3)	0.71004 (7)	0.0398 (4)	
N4	0.64043 (10)	1.1311 (3)	0.61096 (7)	0.0394 (4)	
H4	0.6690	1.2476	0.6036	0.047*	
N5	0.55420 (10)	0.8603 (3)	0.59990 (7)	0.0416 (4)	
H5A	0.5180	0.7738	0.5844	0.050*	
O1	0.40025 (13)	0.7072 (3)	0.55042 (8)	0.0729 (5)	
O2	0.37421 (11)	0.3460 (3)	0.50891 (7)	0.0669 (5)	
O3	0.46938 (10)	0.3929 (4)	0.58895 (7)	0.0720 (5)	
O4	0.53719 (10)	0.2035 (3)	0.79461 (6)	0.0519 (4)	
O5	0.63123 (10)	0.4573 (3)	0.84146 (6)	0.0547 (4)	
O6	0.62452 (10)	0.0766 (3)	0.87476 (7)	0.0567 (4)	
O7	0.28741 (10)	0.4810 (2)	0.40726 (7)	0.0553 (4)	
H1W	0.2375	0.4616	0.3992	0.083*	
H2W	0.2988	0.4323	0.4403	0.083*	
S1	0.57997 (3)	0.26166 (8)	0.84914 (2)	0.04219 (17)	
S2	0.39453 (3)	0.47017 (9)	0.56027 (2)	0.04297 (17)	

### Atomic displacement parameters (Å^2^)

**Table:**

	*U*^11^	*U*^22^	*U*^33^	*U*^12^	*U*^13^	*U*^23^
C1	0.0338 (9)	0.0381 (10)	0.0317 (9)	0.0065 (8)	0.0063 (8)	0.0004 (7)
C2	0.0416 (11)	0.0517 (12)	0.0336 (10)	0.0055 (9)	−0.0047 (8)	−0.0050 (9)
C3	0.0328 (9)	0.0382 (9)	0.0316 (9)	0.0051 (7)	0.0052 (7)	−0.0012 (7)
C4	0.0346 (9)	0.0406 (10)	0.0275 (9)	0.0017 (8)	0.0024 (7)	0.0000 (7)
C5	0.0521 (12)	0.0470 (11)	0.0326 (10)	−0.0033 (9)	−0.0002 (9)	0.0057 (8)
C6	0.0648 (17)	0.112 (2)	0.0718 (19)	−0.0161 (16)	0.0225 (15)	0.0054 (17)
C7	0.0492 (13)	0.0741 (16)	0.0503 (13)	−0.0088 (12)	0.0086 (10)	0.0015 (12)
C8	0.0550 (14)	0.0653 (15)	0.0614 (15)	0.0093 (12)	0.0104 (12)	−0.0015 (12)
C9	0.0560 (13)	0.0478 (12)	0.0531 (13)	0.0019 (10)	0.0022 (11)	0.0063 (10)
C10	0.0415 (11)	0.0442 (11)	0.0372 (10)	−0.0045 (9)	−0.0008 (8)	0.0013 (8)
C11	0.0584 (14)	0.0510 (13)	0.0639 (15)	0.0063 (11)	0.0151 (12)	0.0119 (11)
C12	0.0716 (17)	0.0554 (14)	0.0692 (16)	−0.0057 (12)	0.0161 (13)	0.0190 (12)
C13	0.0567 (15)	0.096 (2)	0.0615 (16)	0.0150 (14)	0.0110 (13)	−0.0165 (14)
C14	0.0411 (11)	0.0591 (13)	0.0408 (11)	0.0044 (10)	0.0006 (9)	−0.0061 (10)
C15	0.0678 (16)	0.0438 (12)	0.0752 (17)	0.0182 (11)	0.0124 (13)	0.0016 (11)
C16	0.0696 (16)	0.0404 (11)	0.0687 (16)	0.0089 (11)	0.0183 (13)	0.0168 (11)
C17	0.0373 (10)	0.0396 (10)	0.0354 (10)	0.0028 (8)	0.0009 (8)	0.0067 (8)
C18	0.0631 (14)	0.0431 (11)	0.0616 (14)	0.0145 (11)	0.0203 (12)	0.0172 (10)
C19	0.0622 (15)	0.0613 (15)	0.0568 (14)	0.0064 (11)	0.0233 (12)	0.0174 (11)
N1	0.0495 (10)	0.0433 (9)	0.0364 (9)	−0.0034 (8)	0.0008 (7)	0.0074 (7)
N2	0.0455 (9)	0.0448 (9)	0.0286 (8)	0.0059 (7)	0.0007 (7)	0.0048 (7)
N3	0.0354 (9)	0.0472 (9)	0.0368 (9)	0.0007 (7)	−0.0010 (7)	−0.0037 (7)
N4	0.0404 (9)	0.0411 (9)	0.0367 (9)	−0.0048 (7)	0.0047 (7)	0.0043 (7)
N5	0.0485 (10)	0.0443 (9)	0.0319 (8)	−0.0097 (8)	−0.0050 (7)	0.0037 (7)
O1	0.0845 (13)	0.0551 (10)	0.0795 (13)	−0.0160 (9)	0.0234 (10)	0.0090 (9)
O2	0.0717 (11)	0.0869 (12)	0.0421 (9)	−0.0227 (10)	0.0037 (8)	−0.0136 (8)
O3	0.0437 (9)	0.1191 (16)	0.0532 (10)	0.0023 (10)	−0.0032 (8)	−0.0017 (10)
O4	0.0592 (9)	0.0593 (9)	0.0374 (8)	−0.0005 (7)	0.0032 (7)	0.0043 (7)
O5	0.0508 (9)	0.0690 (10)	0.0443 (9)	−0.0112 (7)	0.0028 (7)	0.0169 (7)
O6	0.0532 (9)	0.0628 (10)	0.0543 (9)	0.0222 (8)	0.0087 (7)	0.0125 (7)
O7	0.0526 (9)	0.0561 (9)	0.0571 (9)	−0.0114 (7)	0.0004 (8)	0.0093 (7)
S1	0.0414 (3)	0.0495 (3)	0.0358 (3)	0.0052 (2)	0.0047 (2)	0.0099 (2)
S2	0.0426 (3)	0.0509 (3)	0.0354 (3)	−0.0092 (2)	0.0005 (2)	0.0004 (2)

### Geometric parameters (Å, °)

**Table:**

C1—N1	1.311 (2)	C13—C14	1.507 (3)
C1—N2	1.356 (2)	C13—H13A	0.9600
C1—C4	1.410 (3)	C13—H13B	0.9600
C2—N3	1.299 (3)	C13—H13C	0.9600
C2—N2	1.357 (3)	C14—C15	1.361 (3)
C2—H2	0.9300	C14—C19	1.377 (3)
C3—N3	1.352 (2)	C15—C16	1.379 (3)
C3—N4	1.365 (2)	C15—H15	0.9300
C3—C4	1.374 (3)	C16—C17	1.379 (3)
C4—N5	1.376 (2)	C16—H16	0.9300
C5—N4	1.320 (3)	C17—C18	1.368 (3)
C5—N5	1.321 (3)	C17—S1	1.760 (2)
C5—H5	0.9300	C18—C19	1.382 (3)
C6—C7	1.513 (3)	C18—H18	0.9300
C6—H6A	0.9600	C19—H19	0.9300
C6—H6B	0.9600	N1—H1A	0.8602
C6—H6C	0.9600	N1—H1B	0.8608
C7—C12	1.374 (4)	N2—H2A	0.8595
C7—C8	1.380 (4)	N4—H4	0.8601
C8—C9	1.385 (3)	N5—H5A	0.8592
C8—H8	0.9300	O1—S2	1.4513 (18)
C9—C10	1.381 (3)	O2—S2	1.4237 (16)
C9—H9	0.9300	O3—S2	1.4506 (17)
C10—C11	1.383 (3)	O4—S1	1.4576 (16)
C10—S2	1.767 (2)	O5—S1	1.4569 (16)
C11—C12	1.375 (3)	O6—S1	1.4498 (15)
C11—H11	0.9300	O7—H1W	0.8376
C12—H12	0.9300	O7—H2W	0.8259
			
N1—C1—N2	120.98 (17)	C15—C14—C19	117.6 (2)
N1—C1—C4	126.53 (17)	C15—C14—C13	121.8 (2)
N2—C1—C4	112.49 (17)	C19—C14—C13	120.6 (2)
N3—C2—N2	125.47 (18)	C14—C15—C16	122.3 (2)
N3—C2—H2	117.3	C14—C15—H15	118.9
N2—C2—H2	117.3	C16—C15—H15	118.9
N3—C3—N4	126.33 (17)	C17—C16—C15	119.6 (2)
N3—C3—C4	126.72 (17)	C17—C16—H16	120.2
N4—C3—C4	106.96 (16)	C15—C16—H16	120.2
C3—C4—N5	106.63 (16)	C18—C17—C16	119.0 (2)
C3—C4—C1	119.08 (17)	C18—C17—S1	120.36 (16)
N5—C4—C1	134.12 (18)	C16—C17—S1	120.54 (16)
N4—C5—N5	110.20 (17)	C17—C18—C19	120.4 (2)
N4—C5—H5	124.9	C17—C18—H18	119.8
N5—C5—H5	124.9	C19—C18—H18	119.8
C7—C6—H6A	109.5	C14—C19—C18	121.2 (2)
C7—C6—H6B	109.5	C14—C19—H19	119.4
H6A—C6—H6B	109.5	C18—C19—H19	119.4
C7—C6—H6C	109.5	C1—N1—H1A	120.0
H6A—C6—H6C	109.5	C1—N1—H1B	120.0
H6B—C6—H6C	109.5	H1A—N1—H1B	120.0
C12—C7—C8	117.7 (2)	C1—N2—C2	124.11 (16)
C12—C7—C6	120.5 (2)	C1—N2—H2A	117.9
C8—C7—C6	121.8 (2)	C2—N2—H2A	118.0
C7—C8—C9	121.4 (2)	C2—N3—C3	112.03 (17)
C7—C8—H8	119.3	C5—N4—C3	108.31 (16)
C9—C8—H8	119.3	C5—N4—H4	125.9
C10—C9—C8	119.7 (2)	C3—N4—H4	125.8
C10—C9—H9	120.1	C5—N5—C4	107.90 (16)
C8—C9—H9	120.1	C5—N5—H5A	126.1
C9—C10—C11	119.4 (2)	C4—N5—H5A	126.0
C9—C10—S2	121.40 (16)	H1W—O7—H2W	110.9
C11—C10—S2	119.21 (17)	O6—S1—O5	112.91 (10)
C12—C11—C10	119.7 (2)	O6—S1—O4	112.80 (10)
C12—C11—H11	120.1	O5—S1—O4	111.18 (9)
C10—C11—H11	120.1	O6—S1—C17	106.48 (9)
C7—C12—C11	122.0 (2)	O5—S1—C17	106.79 (10)
C7—C12—H12	119.0	O4—S1—C17	106.13 (9)
C11—C12—H12	119.0	O2—S2—O3	112.64 (12)
C14—C13—H13A	109.5	O2—S2—O1	114.04 (12)
C14—C13—H13B	109.5	O3—S2—O1	109.38 (13)
H13A—C13—H13B	109.5	O2—S2—C10	107.32 (10)
C14—C13—H13C	109.5	O3—S2—C10	105.60 (10)
H13A—C13—H13C	109.5	O1—S2—C10	107.35 (10)
H13B—C13—H13C	109.5		
			
N3—C3—C4—N5	−179.98 (17)	C13—C14—C19—C18	178.4 (2)
N4—C3—C4—N5	−0.4 (2)	C17—C18—C19—C14	0.6 (4)
N3—C3—C4—C1	−4.1 (3)	N1—C1—N2—C2	178.16 (18)
N4—C3—C4—C1	175.47 (16)	C4—C1—N2—C2	−1.0 (3)
N1—C1—C4—C3	−175.61 (18)	N3—C2—N2—C1	−1.6 (3)
N2—C1—C4—C3	3.5 (2)	N2—C2—N3—C3	1.3 (3)
N1—C1—C4—N5	−1.2 (3)	N4—C3—N3—C2	−177.93 (18)
N2—C1—C4—N5	177.89 (19)	C4—C3—N3—C2	1.6 (3)
C12—C7—C8—C9	0.3 (4)	N5—C5—N4—C3	−0.2 (2)
C6—C7—C8—C9	−179.4 (2)	N3—C3—N4—C5	179.97 (18)
C7—C8—C9—C10	0.0 (4)	C4—C3—N4—C5	0.4 (2)
C8—C9—C10—C11	0.0 (3)	N4—C5—N5—C4	0.0 (2)
C8—C9—C10—S2	178.90 (18)	C3—C4—N5—C5	0.3 (2)
C9—C10—C11—C12	−0.3 (4)	C1—C4—N5—C5	−174.7 (2)
S2—C10—C11—C12	−179.2 (2)	C18—C17—S1—O6	−26.8 (2)
C8—C7—C12—C11	−0.6 (4)	C16—C17—S1—O6	157.08 (19)
C6—C7—C12—C11	179.1 (3)	C18—C17—S1—O5	−147.66 (19)
C10—C11—C12—C7	0.6 (4)	C16—C17—S1—O5	36.2 (2)
C19—C14—C15—C16	0.2 (4)	C18—C17—S1—O4	93.6 (2)
C13—C14—C15—C16	−179.4 (2)	C16—C17—S1—O4	−82.5 (2)
C14—C15—C16—C17	1.4 (4)	C9—C10—S2—O2	−106.24 (19)
C15—C16—C17—C18	−2.0 (4)	C11—C10—S2—O2	72.7 (2)
C15—C16—C17—S1	174.2 (2)	C9—C10—S2—O3	133.38 (19)
C16—C17—C18—C19	1.0 (4)	C11—C10—S2—O3	−47.7 (2)
S1—C17—C18—C19	−175.2 (2)	C9—C10—S2—O1	16.7 (2)
C15—C14—C19—C18	−1.2 (4)	C11—C10—S2—O1	−164.35 (19)

### Hydrogen-bond geometry (Å, °)

**Table:**

*D*—H···*A*	*D*—H	H···*A*	*D*···*A*	*D*—H···*A*
N1—H1A···O4	0.86	1.95	2.813 (2)	178
N1—H1B···O3	0.86	1.97	2.805 (2)	163
N2—H2A···O5	0.86	1.84	2.694 (2)	178
N4—H4···O7^i^	0.86	1.80	2.653 (2)	170
N5—H5A···O1	0.86	2.09	2.884 (3)	152
N5—H5A···O3	0.86	2.43	3.149 (3)	141
O7—H1W···O6^ii^	0.84	1.93	2.762 (2)	176
O7—H2W···O2	0.83	2.04	2.815 (2)	156

Symmetry codes: (i) −*x*+1, −*y*+2, −*z*+1; (ii) *x*−1/2, −*y*+1/2, *z*−1/2.

## References

[bb1] Barral, K., Priet, S., Sire, J., Neyts, J., Balzarini, J., Canard, B. & Alvarez, K. (2006). *J. Med. Chem* **49**, 7799-7806. 10.1021/jm060030y 17181162

[bb2] Bernstein, J., Davis, R. E., Shimoni, L. & Chang, N.-L. (1995). *Angew. Chem. Int. Ed. Engl.* **34**, 1555–1573.

[bb3] Bruker (2006). *APEX2*, *SAINT* and *SADABS* Bruker AXS Inc., Madison, Wisconsin, USA.

[bb4] Burnett, M. N. & Johnson, C. K. (1996). *ORTEPIII* Report ORNL-6895. Oak Ridge National Laboratory, Tennessee, USA.

[bb5] Etter, M. C. (1990). *Acc. Chem. Res.* **23**, 120–126.

[bb6] Farrugia, L. J. (1997). *J. Appl. Cryst.* **30**, 565.

[bb7] Pearce, L., Prout, C. K. & Watkin, D. J. (2000). *CAMERON* Chemical Crystallography Laboratory, University of Oxford, England.

[bb8] Sheldrick, G. M. (2008). *Acta Cryst.* A**64**, 112–122. 10.1107/S0108767307043930 18156677

[bb9] Sridhar, B. & Ravikumar, K. (2007). *Acta Cryst.* C**63**, o415–o418. 10.1107/S0108270107024493 17609575

[bb10] Sridhar, B., Ravikumar, K. & Varghese, B. (2009). *Acta Cryst.* C**65**, o202–o206. 10.1107/S0108270109011780 19407416

[bb11] Xing, D., Tan, X., Chen, X. & Bu, Y. (2008). *J. Phys. Chem. A*, **112**, 7418-7425. 10.1021/jp800256v 18646734

